# Is Injury an Occupational Hazard for Horseracing Staff?

**DOI:** 10.3390/ijerph19042054

**Published:** 2022-02-12

**Authors:** Emma Davies, Will McConn-Palfreyman, John K. Parker, Lorna J. Cameron, Jane M. Williams

**Affiliations:** 1 Equestrian Performance Research Centre, Hartpury University, Gloucestershire GL19 3BE, UK; lorna.cameron@hartpury.ac.uk (L.J.C.); jane.williams@hartpury.ac.uk (J.M.W.); 2 SportScotland Institute of Sport, Stirling FK9 5PH, UK; will.mcconn@gmail.com; 3 Sport and Exercise Research Centre, Hartpury University, Gloucestershire GL19 3BE, UK; john.parker@hartpury.ac.uk

**Keywords:** occupational injury, injury reporting, safety at work, psychosocial risk factors, injury management, workforce retention, health, horseracing

## Abstract

Occupational health is a key priority for the horseracing industry, yet little research on occupational injuries exists. This study investigated the prevalence and the effect of injury in British horseracing staff during a 12-month period. An online retrospective survey was answered by 352 participants, identifying self-reported injury prevalence, injury management practices and attitudes towards workplace injury reporting. Chi Squared tests for independence were undertaken. A total of 310 (88.1%) staff reported injuries; risk factors for injury type included self-perceived job security, working hours, and perceived job control. Physical limitations, loss of confidence, workplace changes, and lifestyle implications were reported as consequences of injury. A total of 75.3% (*n* = 134) of staff were likely to seek time-off following fractures, but only 48.6% (*n* = 86) would take time-off for concussion. Attitudes towards injury management were influenced by financial circumstances, perceived staff shortages, previous injury experiences, and perceived employer expectations. The high self-reported injury prevalence could result in decreased workforce efficiency, poor physical health, and negative implications on retention and career longevity. The perception of invisible injuries, i.e., concussion, and subsequent management, should be of immediate concern to racing organizations. This paper identifies recommendations to enhance the safety and wellbeing of horseracing staff.

## 1. Introduction

Stable staff, also known as racing grooms, have a multifaceted role within the horseracing industry, acting as care givers, skilled athletes, and equine experts [1,2], which often results in a role with inherently high emotional, physical, and mental demands [3], and subsequently high injury rates amongst staff [2,4,5]. Core staff includes track riders (riding only role), racing grooms (training yards) and stud grooms (stud and breeding yards) who are involved in 82% of all reported accidents [4], and often present differing injury profiles to jockeys [5]. Racing and stud grooms have increased manual labour and equine contact hours in comparison to jockeys, which may explain the differences in injury profile, reflecting the complex nature of the role of horseracing grooms. Whilst the horse is a significant risk factor for injury in both ridden and handling roles (see [6] for full review), additional occupational risks may exist for stable staff that are not seen in jockeys [2,4] despite both working in high-risk occupations [7]. Previous research and anecdotal reports highlight that whilst staff experience high levels of injury, the likelihood of reporting injuries, seeking time-off or treatment, or resting during recovery is low [2,4,8,9]. Recent research in horseracing noted a disregard for work-based injuries reported by those staff working in direct contact with horses [10] and a culture of presenteeism, turning up to work when injured or unwell [3,9,11,12]. Employees who ignore their own health needs may experience higher levels of physical and mental stress, which can increase the risk of occupational injury and may impact the efficiency of the workforce [13,14,15]. Previous workplace stress research has reported increased mistakes due to lack of concentration or poor decision-making, slower completion of tasks and increased absenteeism [13,14,15], which could have an impact on the demands placed upon other staff, as well as potentially on the welfare of the racehorses if standards of care are not maintained [16,17,18]. The social demand for strict welfare standards in horseracing has led to a culture of ‘putting the horse first’ [19]. These priorities have resulted in an industry which maintains that it has some of the highest welfare standards in the equestrian sector, that has continuously worked to promote the application of scientific evidence-based training and welfare principles and to upskill staff to maintain those standards [17,19,20]. However, the ‘horse first’ culture may have inadvertently reinforced a workforce who deprioritize their own health and wellbeing to care for the horse, which has been previously reported in other animal care sectors [18].

Employers have a legal obligation to ensure the health, safety, and welfare of their employees, which includes occupational health services (OHS) [21,22,23]. Globally, there are conflicting legal requirements for OHS provision; whilst European organizations are legally obligated to provide OHS services for staff, there are no corresponding legal requirements for employers in Britain and Ireland [23]. Previous research has identified that where OHS services are provided or actively promoted by the employer, there is a decrease in injury risk within that organization, alongside improved compliance with health and safety protocols [21,22,24]. Research suggests only 51% of British employees have access to OHS, including a mixture of NHS provision and private services [25]. The International Labor Organization (ILO) Occupational Health Services Convention (No. 161) defined OHS as:

“…*services entrusted with essentially preventive functions and responsible for advising the employer, the workers and their representatives in the undertaking on the requirements for establishing and maintaining a safe and healthy working environment which will facilitate optimal physical and mental health in relation to work and the adaptation of work to the capabilities of workers in the light of their state of physical and mental health*…”

[23]

Within British horseracing, OHS is provided through Racing Welfare, a charity dedicated to the provision of human welfare services for the industry and enhancing the wellbeing of horseracing staff [26]. Whilst available for all staff, the OHS provided by Racing Welfare are voluntary, and engagement relies largely on employer promotion [22,23]. In addition, the memorandum of agreement between the National Trainers Federation (NTF) and the National Association of Racing Staff (NARS), items 16 and 17, identifies absence from work, sickness pay, and the Racing Industry Accident Benefit Scheme (RIABS), which is a mandatory insurance scheme for staff who are off work following accidental injury arising from carrying out duties at work [1]. These contractual requirements are in line with the U.K. national legislative framework requiring all British employers to exercise a duty of care to employees at work, and statutory accident insurance [23].

The unique nature of the role of stable staff, along with the cultural considerations of horseracing as a competitive sport and industry (see [16] for full review), poses a novel situation within which to consider the effects of injury on the workforce. The result of such research could have important implications for increasing knowledge of injury causation and psychosocial risk factors within horseracing staff, as well as the design and training of targeted interventions for employers and employees to optimize workplace health and safety, and to enhance staff education around injury management. The aim of this exploratory study was therefore to investigate the prevalence and subsequent effects of injury in British horseracing grooms during a 12-month period. To approach this overarching aim, 4 specific research objectives were addressed: (1) to identify prevalence of self-reported injury in horseracing stud and stable staff within a 12-month period; (2) to determine risk factors for racing staff injuries; (3) to investigate how horseracing grooms manage injury in the workplace; (4) to identify staff’s attitudes towards injury and injury reporting behaviours.

## 2. Materials and Methods

### 2.1. Design

A descriptive, cross-sectional, retrospective online survey design was used in this study. The use of online surveys allows for interactions with a more diverse respondent group whilst obtaining a large sample at the convenience of the researcher and participant [27]. Research has questioned the validity of retrospective data collection in reflecting on experiences of injury [28], however, equestrian athletes have been found to recall injury experiences and reflect on positive and negative emotions successfully [29,30], whilst Southwick et al. [31] suggests that memory recall is remarkably accurate for injury experiences. Furthermore, horseracing staff are required to identify injuries within the yard accident book at the time of injury [20], which may allow for more accurate memory recall [28]. The survey was piloted using a purposeful sample (*n* = 10) of local horseracing staff.

### 2.2. Participants and Recruitment

Following institutional ethical approval by the Hartpury University Human Research Ethics Committee (approval number ETHICS2019-67) and informed consent, eligible British horseracing staff (*n =* 352) voluntarily provided unidentifiable online survey data. Participants were eligible if they were over 18 years old and had been employed in the British horseracing industry for a minimum of 12 months in a horse-handling related role, including but not limited to: stud grooms, racing grooms, assistant trainer, trainer, or work rider. Participants must have been working for a minimum of 12 months in the industry to ensure they could provide 12 months of injury data for the survey aims. Participants working in administrative roles, who had been employed in racing for less than 12 months or were not actively employed by the racing industry at the time of the survey were excluded.

In 2018, there were 6734 registered racing employees, 4428 of which were full-time, working in over 550 licensed race yards responsible for the care and training of 23,599 horses in the UK [20]. Previous research has utilized samples ranging from 119 employees [8] to 2293 employees [4], although the latter study reported only 49% (*n* = 1124) of these involved injury incidents. Recruitment was achieved through personal and organisational industry contacts, collaborating partners and social media groups/pages to recruit participants who meet inclusion/exclusion criteria [32]. Engagement with surveys in recent years has been considered a strength of employees within the racing industry [33], and similar methods have been utilized to gain prior injury and mental health data from stable staff with substantial sample sizes gathered [2,4,10]. The sample was purposive and therefore not representative of the wider horseracing population, however, potential respondent bias was minimized by utilizing a wide range of online sites to recruit participants, such as Facebook, Twitter, Instagram and through direct communication with the industry via organisations and member bodies (emails and newsletters) [27].

### 2.3. Measures and Procedure

The online survey (See Supplementary File S1) was conducted using Qualtrics CoreXM 2021 survey software. Participants completed 24 closed and 2 open questions, which took approximately 11 min to complete. Questions were designed by the research team, informed by Speed and Anderson [7], Filby and Jackson [8], and Filby, Jackson and Turner [4], to investigate injury prevalence, risk factors and injury reporting behaviours within horseracing staff, and covered four areas of significance: employee demographics, employment characteristics, injury characteristics, and injury management, including coping strategies. The justification for the inclusion of these areas is provided in Table 1.

Prior to starting the questionnaire, participants were given information pertaining to their data protection rights, risks and benefits, and withdrawal procedures before being asked to consent to the study; no identifiable data were collected. The survey was live from 7 January 2021, following an industry sector launch, and closed on 28 February 2021. All surveys were used in analysis, and data were analysed on a question-by-question basis.

### 2.4. Data Analysis

Data were exported from QualtricsXM (Qualtrics, Seattle, WA, USA) to Microsoft Excel (Office 365, (Microsoft Corporation, Washington, DC, USA)). Frequency analysis was used to assess injury prevalence, reporting behaviour and pain management practices. Following assumption testing for normality, data were analyzed using IMB Statistical Product and Service Solutions (SPSS) software version 26 (IBM, New York, NY, USA). Chi Squared tests for independence were used to identify associations; significance *p* < 0.05.

Content analysis identified 8 higher order themes across the 2 open-ended questions [41,42], which investigated how injury had affected staff within the last 12 months of employment, as well as to explain why staff were likely to demonstrate certain behaviour relative to seeking medical attention and reporting injuries in the workplace. The first author (ED) familiarised herself with the textual data and engaged in constant comparison to chunk the text into segments [43]. During the organisation phase, an inductive approach was used to generate and apply descriptive codes to the comments according to the content [44,45]. Related codes were combined to produce sub-categories and summarised into key categories based on the content [46]. The analysis was repeated across both open-ended questions, and categories were verbally discussed with the investigation team to ensure clarity of concepts, and validity of identified codes [43].

## 3. Results

### 3.1. Demographics

A total of 352 respondents (±5.1% Margin of Error for the British stable staff population (~7000) at 95% CI) gave data on injuries obtained within the last 12 months whilst working in a horse-handling role in British horseracing. Following the initial questions on injury, a total of 154 participants withdrew from the survey (43.75%), resulting in demographic data on 198 respondents (95% CI, ±7% Margin of Error). A total of 80.3% (*n* = 155) of respondents were female, 19.2% were male (*n* = 37) with six respondents not stating their gender. Participants ranged in age from 18 to 73 years, with an average age of 34.22 ± 12.75 years old.

#### 3.1.1. Employment Characteristics

Of the 196 respondents who gave information on their employment, 56.1% (*n* = 110) were employed full-time, 20.4% (*n* = 40) part-time and 14.3% (*n* = 28) were self-employed staff. The remaining 9.2% (*n* = 18) selected option ‘other’, which consisted of casual, unemployed, apprentice, those on sick leave and directors of limited companies. Employment sectors were broken down into Code as per Table 2, with most staff working in both flat and jump racing. The category of “other” predominately listed stud as the code.

When asked to identify their role in the horseracing industry, 48.5% identified as a rider/groom (racing groom), 4.6% were trainers, 5.6% travelling grooms, 11.2% identified as a yard person, whilst 10.2% were working in a supervisory role, and 10.2% were stud hands. Finally, staff were asked to identify the number of years they had been working in the horseracing industry (Table 3).

#### 3.1.2. Occupational Attitudes

Most staff reported regularly working 8–9 h per day as part of their role (33.67%, *n* = 66), however, 6.1% (*n* = 12) staff reported working over 12 h a day (Table 4). When asked about whether they were satisfied with their working hours, 69.4% (*n* = 136) were satisfied with their current working hours. A further 5.1% (*n* = 10) would have liked to work more hours, whilst 25.5% (*n* = 50) reported wanting to work less hours than they currently do.

Staff were asked to rate the level of control they had within the workplace over daily tasks on a scale from 1 (no control) to 5 (complete control). Of the 196 respondents, 36.2% (*n* = 71) reported having a lot, or complete control over their daily tasks at work, 28.6% (*n* = 56) identified a moderate amount of control, whilst 35.5% (*n* = 69) reported little to no control over daily tasks. When asked about perceived job security in the next 12 months, 78.6% (*n* = 154) were positive about the security of their role, whilst a further 13.3% (*n* = 26) were unsure, and only 8.2% (*n* = 16) perceived it unlikely to still have their role in the next 12 months.

### 3.2. Injuries

A total of 352 respondents provided data on injuries obtained within the last 12 months whilst working in a horse-handling role in British horseracing. A total of 1164 injuries were reported for 310 participants, with a further 42 people (11.9%) reporting obtaining no injuries in the last 12 months. As an approximation of the eligible population (~7000), incidence rate for injury is estimated at 0.166 injuries/person, with staff in this survey reporting 3.30 injuries/person in the 12-month period surveyed. The most common injuries reported were bruises (*n* = 283, 23.47%), lower back pain (*n* = 175, 14.5%), muscle strain (*n* = 163, 13.5%), upper back/neck pain (*n* = 105, 8.7%), lacerations (*n* = 72, 5.97%), tendon/ligament damage (*n* = 63, 5.2%) and suspected concussion (*n* = 62, 5.1%). A full breakdown of injuries can be seen in Table 5.

There were no significant associations (*p* > 0.05) between whether a staff member was injured in the last 12 months and any of the following risk factors: employment status, job role, code, years working in industry, working hours, attitude to working hours, perception of job security or perception of job control. However, when categorized by type of injury, significant associations between injury type reported and several occupational risk factors (job control, perception of job security, hours worked, satisfaction with working hours) were identified (*p* < 0.05).

#### 3.2.1. Job Control

Significant associations were found between perception of control of daily tasks at work (job control) and the types of injuries reported in the last 12 months. Staff were statistically more likely to have reported chronic musculoskeletal injuries if they had little to no control over daily tasks: muscle strain (*p* = 0.0004), upper back/neck pain (*p* = 0.045), lower back pain (*p* = 0.033) or nerve damage (*p* = 0.005). In addition, staff who had reported no injuries in the last 12 months were statistically more likely to have control over all their daily tasks in the workplace (*p* = 0.001).

#### 3.2.2. Perceived Job Security

There were significant associations between perceived job security and the types of injury a staff member had experienced in the last 12 months. Staff were statistically more likely to report feeling insecure in their future employment if they had experienced a leg/foot fracture (*p* = 0.006), bruised or fractured ribs (*p* = 0.032), diagnosed concussion (*p* = 0.046) or spinal fractures (*p* = 0.004) in the last 12 months.

#### 3.2.3. Working Hours

There were also significant associations between the number of hours staff worked per day (on average) and the types of injuries a staff member had experienced in the last 12 months. Staff working 8–9 h/day were at the most risk of chronic musculoskeletal injuries, including muscle strain (*p* = 0.042) and soft tissue injuries (*p* = 0.045), whilst those working 10–11 h/day were most likely to report lower back pain (*p* = 0.014). Significant associations also occurred between whether staff were satisfied with the hours they worked, and the types of injuries reported. Those staff who stated they worked too many hours and would prefer to work less, were statistically more likely to report musculoskeletal injuries including sprained wrist (*p* = 0.011), sprained ankle (*p* = 0.0004), muscle strain (*p* = 0.012), lower back pain (*p* = 0.0004), nerve damage (*p* = 0.003) or fractured leg/foot (*p* = 0.0004). In addition, staff who were satisfied with the hours they worked were less likely to report bruises obtained from the workplace (*p* = 0.003).

Following the initial questions reporting injuries in the last 12 months, 154 people voluntarily withdraw from the survey (43.75%), resulting in data for 198 participants on injury management, injury attitudes and behaviours surrounding injury in the workplace.

### 3.3. Injury Management

Participants were asked to report on how injury had affected their working life through a series of questions. When asked whether their injuries had resulted in adaptations made to their working environment (*n* = 198), 51.52% (*n* = 102) respondents identified that no adaptations had been made. Of those who reported workplace adjustments (*n* = 96, 48.48%), 158 adaptations were identified, averaging 1.65 adaptations made on average per person. These are listed in the Table 6 below:

**Table 6:** Adjustments made at work due to injuries in the last 12 months.

Adaptation	Number of Responses	Percentage (%)
Stop Work	44	45.83
Reduced Duties (non-riding)	26	27.08
Not allowed to ride	25	26.04
Decreased hours	16	16.67
Reduced days per week	16	16.67
Restrictions on riding	14	14.58
Rest breaks	7	7.29
Other	10	10.42

**Figure 1:**
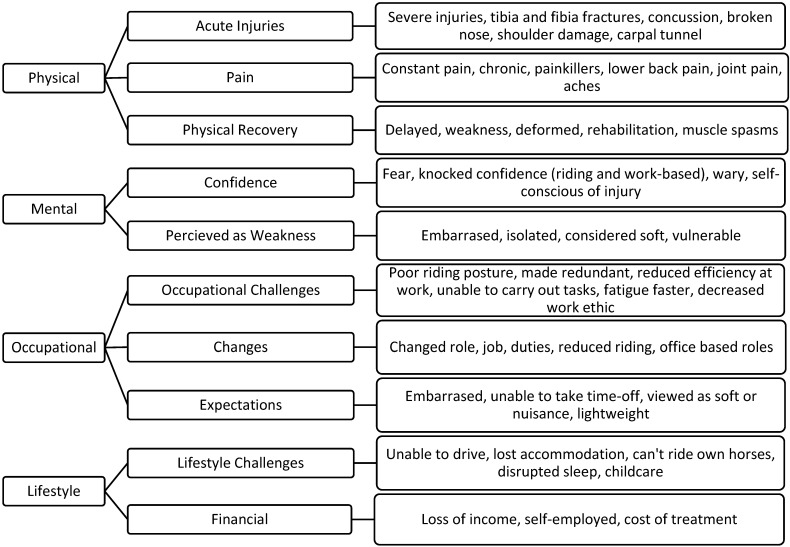
The effects of injury on racing staff.

Using an open-ended question, participants were also asked to identify how injury had affected them overall in the last 12 months. Four main themes were identified, surrounding physical, mental, occupational and lifestyle impacts (Figure 1).

Racing staff were asked to report on their use of over-the-counter pain medication (i.e., ibuprofen or paracetamol) to manage daily tasks at work. Of the 200 respondents who commented on pain management use, over half (*n* = 104, 52%) took pain killers at least once a week to manage daily tasks at work, with the most common answer once per week (*n* = 36, 18%). A total of 10% (*n* = 20) of respondents took pain killers daily, whilst a further 8% (*n* = 16) took them more than once per day.

When asked to identify whether they used any medication or drugs to manage physical pain at work (322 responses), 10% (*n* = 33) identified that they had not used any substances in the last 12 months to manage physical pain at work. Over-the-counter medication was reported to be used in 137 responses (42.55%), whilst 20.5% of respondents (*n* = 66) reported the use of prescription pain killers (with a specific prescription).

#### Support

Racing staff were asked to report on the use and perceptions of common support networks and services during periods of injury. Participants were divided on whether work friends, parents, their employer, and their spouse were helpful during injury, with 41.11% of staff reporting their employer as unhelpful during periods of injury (Table 7).

Not using identified support services during periods of injury was common (44.44%, *n* = 100), whilst those who did typically used either the National Health Service (NHS) (26.67%, *n* = 60) or Racing Welfare (10.22%, *n* = 23) for support and advice.

### 3.4. Attitudes to Injury Reporting and Injury Management Behaviours

For four common injury types (any fracture, all other musculoskeletal injuries, suspected or diagnosed concussion, and any other head injuries), racing staff were asked to rate their likelihood of seeking medical attention beyond immediate first aid, reporting the injury to their employer, and taking time-off because of said injury.

#### 3.4.1. Seeking Medical Attention beyond First Aid

A total of 96.07% (*n* = 171) of racing staff were likely to seek medical attention for a fracture, 91.67% (*n* = 162) were likely following any head injury, 66.1% (*n* = 117) were likely to seek medical attention following a suspected or diagnosed concussion and 64.4% (*n* = 114) were likely following any other musculoskeletal injuries. Racing grooms were less likely to seek medical attention for head injuries than other industry job roles (*p* = 0.001). Interestingly, female staff were less likely to seek medical attention beyond first aid for musculoskeletal injuries (*p* = 0.026) and head injuries (*p* = 0.008) than male staff.

#### 3.4.2. Reporting to an Employer

Whilst 94.3% (*n* = 182) of staff were likely to report a fracture to their employer, and 91.67% (*n* = 176) were likely to report a head injury, only 77.09% (*n* = 148) of staff were likely to report a diagnosed or suspected concussion to their employer. For musculoskeletal injuries, 75.52% (*n* = 145) of staff would report those injuries to their employer. Job role was significantly associated with likelihood of reporting injuries to an employer. Racing grooms, riders and yard staff were all less likely to report fractures (*p* = 0.0004), concussion (*p* = 0.030), and head injuries (*p* = 0.0004) to their employer than other staff roles. Flat racing staff were less likely to report fractures (*p* = 0.0004), concussion (*p* = 0.009), and head injuries (*p* = 0.0004) than other groups. Point-to-point staff were also less likely to report fractures than other groups (*p* = 0.0004). Finally, female staff were less likely to report musculoskeletal injuries to their employer than male staff (*p* = 0.037).

#### 3.4.3. Taking Time-Off Work Resulting from Injury

Despite higher percentages of staff seeking additional medical attention and reporting to their employer, only 75.3% (*n* = 134) of staff identified that they were likely to take time-off due to a fracture, 83.1% (*n* = 147) were likely to take time-off after any other head injuries. Only 48.6% (*n* = 86) were likely to take time-off work resulting from a suspected or diagnosed concussion, and 44.06% (*n* = 78) from a musculoskeletal injury. There was a significant association between gender and the likelihood of taking time-off for all musculoskeletal injuries (*p* = 0.011), with female staff less likely to take time-off than male staff following injury.

**Figure 2:**
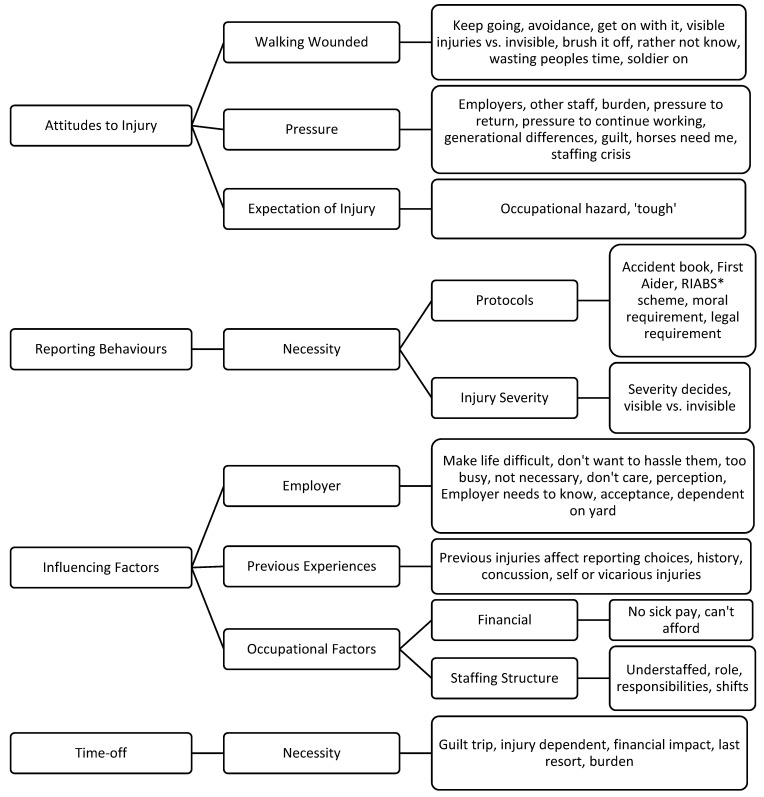
Attitudes and Perceptions of Injury Reporting and Injury Management Behaviour. * RIABS scheme is the Racing Industry Accident Benefit Scheme which provides benefits to eligible persons who are off work following accidental injury arising out of and in the course of employment in the racing industry [47].

Four main themes were identified in response to views on injury reporting and management behaviours: attitudes to injury, reporting behaviours, influencing factors, and time-off (Figure 2).

## 4. Discussion

This exploratory study aimed to investigate the prevalence and effect of injury in British horseracing staff. The results established high self-reported incidents of pain, musculoskeletal injuries, and suspected concussion, with several risk factors for injury type related to occupational demands, such as working hours. When injured, staff typically relied on pain management strategies to continue working, and if adaptations were required at work, these typically involved reduced duties, or restrictions in ridden activities. Horseracing staff were less likely to report or take time-off for invisible injuries, such as concussion or musculoskeletal pain, compared to fractures, and attitudes towards injury reporting and management were influenced by several factors, including financial circumstances, perception of staff shortages, previous injury experiences, and perception of employer and peer expectations.

### 4.1. Injury and Risk Factors

Injury rates were high in racing staff; only 11.9% of participants reported no injuries in the last 12 months. Injury incidence was estimated at 0.166 injuries/person for the population of British horseracing staff (~7000), with staff in this survey reporting 3.30 injuries/person in the 12-month period surveyed. Racing staff have previously reported very high injury incident rates, with over 50% of yards reporting accidents [2,4,8]. Racing staff typically work long, unsocial hours, with 52.53% (*n* = 103) of staff typically working more than 8 h per day. In a recent study, trainers reported long work hours as one of the sources of stress in their profession [9], whilst over 85% of stable staff surveyed in Australia reported working more than 40 h/week, averaging 46 h/week in full-time staff [7]. Mandatory overtime can reduce perceptions of job control, which is a predictor for burnout, and can result in an increased number of sick days for the same injury compared to those staff who did not work overtime [13]. This may be a concern for racing staff who previously reported working overtime or on days off to cover reduced numbers of staff on the yard on race days [17]. The National Association of Racing Staff (NARS) report that no employee should work more than 48 h on average over a 7-day period in Great Britain [48], however, limited research is available to confirm this. Increased hours have been reported to link to higher levels of fatigue and psychological distress, which can increase the risk factor for injury in several occupations, including veterinary, nursing and construction industries [49,50,51]. Care should be taken when considering working hours in relation to job demand in this study, as data collection took place during the 3rd national coronavirus lockdown between January and February 2021 [52]. Whilst many industries were closed during this period due to government-imposed restrictions, previous research from the 1st national lockdown in March 2020 shows that most horseracing staff were likely to be working the same number of hours, with only 32.8% working fewer hours during a COVID-19 lockdown than normal [34]. This suggests that the working behaviours reported in this study are representative of typical occupational demands for horseracing staff.

Very few studies have formally investigated injury incidence or the patterns of injury in stable staff [4,7,8], but the research available suggests the profile of injury for staff is different to that seen in jockeys, who have received greater representation within the empirical literature [5,7,53,54,55]. The most common injuries reported in this study were bruises and lacerations, upper and lower back pain, chronic musculoskeletal issues, and suspected concussion, which aligns with Cowley et al. [5], who found higher rates of back injuries in staff compared to jockeys (16% vs. 9%, respectively). Chronic and overuse injury have also been reported for equestrian athletes in other disciplines, with between 74–96% of riders reporting pain, predominately linked to the neck and back [56,57,58,59]. Occupational demands between Olympic-discipline riders and those working in horseracing could be seen to be similar; long hours and weekend/shift work, workforce instability, and low job control due to strict health and safety requirements of the role, and the requirement to maintain equine welfare standards—all of which increase the risk of injury [2,14,60,61]. Daily repetitive tasks for horseracing grooms, such as sweeping, mucking out or lifting and carrying, are all risk factors for increased neck and back pain [62,63,64,65]. Overuse injuries in horseracing staff could decrease the efficiency of the workforce [13,14,15], and issues of poor horse welfare can also arise when staff are not fully engaged in their daily tasks, a potential consequence of physical and mental fatigue [19].

Several factors influenced the type of injuries that staff were likely to have experienced in a 12-month window, including perceived job control, and working hours. Organisational structure and working conditions have previously been reported as causal factors for injury risk in occupational settings [15]. Staff who felt they had little control over their daily tasks at work were more likely to have experienced musculoskeletal injuries in the last 12 months, whilst those staff who had full control over their job were more likely to report no injuries sustained. Job control is defined as the feeling of autonomy in the workplace, through control over work shift patterns, hours, and responsibility for management and timing of daily tasks and is often limited in high-risk roles due to health and safety [61]. Racing grooms are required to work long hours, with increasing weekend shift work due to the expansion of the fixture list [17]. Anecdotal reports identify struggling to access doctor’s appointments or co-ordinate calendars for off-work activities due to ever changing schedules [2,7,9], which has been exacerbated in recent months by the impact of COVID-19 on racing staff (see [34] for further detail). In addition, staff are required to demonstrate stringent management practices to ensure high standards of horse care and consequently welfare, such as in handling and exercising to avoid equine injury. The rigor of these management practices can result in a perceived loss of job control, which was recently reported by stable staff in an industry study [10]. Research suggests that roles with limited control over daily tasks, and that have increased physiological and psychological demands such as seen for racing staff, can be classified as high strain roles [13]. These highly demanding roles increase physiological arousal that cannot be effectively managed due to limited job control, therefore resulting in internal mental fatigue and physical exhaustion [36,61]. Employees in high-strain occupations may also lack the ability to recover if annual leave or days off are limited, or if off-work situations are directly linked to the job role, i.e., provision of employee housing, as observed in the racing industry [2,61]. The inability to recover can lead to accumulation fatigue, reduced coping mechanisms and subsequent injury from poor decisions [60] which may explain the high injury incidence and the associations seen here between perceived job control and injury type. Previous research has identified that where occupational demands are unable to be alleviated due to health and safety regulations, such as in training yards and studs, individual intervention strategies are an effective method to reduce injury occurrence [64,66]. This may include staff education about early identification of increased muscle tension as a predictor of injury, or management of equipment use in repetitive tasks, such as mucking out, which can reduce the incidence of back pain [64].

In addition, the current study identified that staff working 10–11 h were at increased risk of lower back pain compared to other staff, whilst those working 8–9 h were more likely to experience more musculoskeletal injuries, such as sprains, strains, and muscle pain. Working longer hours has been seen in other industries to increase the risk of injury [14] due to greater levels of physiological and psychological fatigue, combined with a lack of recovery time. Whilst the National Association of Racing Staff (NARS) recommends a 48-h average working week (over a 7-day period), de Castro and Fujishiro [13] identified that working over 40 h per week increases the risk of work-based illnesses, sick days, and back pain. Over half of the participants in this study (52.53%, *n* = 103) were working more than an average 40-h week (typically 8+ h per day), with 6.1% working an average 60-h week, which may explain the increased injury reported in staff working longer hours. Due to the nature of the role in caring for animals as well as the financial challenges trainers face related to recruitment and retention in the current climate [9,17,67,68], recommendations to reduce working hours for stable staff as a method to reduce injury risk are unrealistic. Therefore, other preventative strategies are required to maximise health and safety for racing staff in training and stud yards to counteract the effect of physical fatigue from working longer hours. Whilst longer hours are typically associated with increased stress responses, and higher risk of injury [13,14]; perception of working hours by staff in other vocational sectors, such as nursing and health, or veterinary, has also been shown to influence injury risk [69]. Satisfaction with working hours reduces employee perception of associated stress [69,70], which could reduce the risk of occupational injury, as daily stressors have been found to increase injury risk through altered cognitive function, memory loss, sleep disruptions and impaired relationships [15,71,72]. Racing staff in this survey who wished to work less hours were more likely to report injuries in the last 12 months, which would suggest that work-place satisfaction is a key contributor to injury risk in this population and should be explored further.

Something of note within these data is the unexpectedly high rate of survey drop out (43.75%) after participants reported their personal injury experiences. This finding would suggest that participants who began the survey were willing to report their injuries but chose not to continue the survey when asked to consider the psychosocial factors or wider effects of the injury experienced. It has been suggested that pain is a culturally accepted construct within certain vocations, and thus positively embraced as a sign of success or ‘fitting in’ with the culture [73,74], which could be an explanation for the survey engagement behaviours seen here. Anecdotally, those working in equestrian and horseracing industries may be more likely to verbalize injury as a ‘badge of honour’, something recently identified in McVey [75], who found riders in their ethnographic study were likely to show off injuries as a sign of their commitment to the hard work of owning and riding horses. Dancers have previously reported comparing their injuries to one another with a sense of pride [73], and view pain as a sign of personal improvement [76], whilst female rugby players describe bruises as a sign of physical ability and strength [77]. In addition, farmers identified an honour and prestige attached to continuing to work despite injury [78]. Wider equestrian culture has been shown to take a stoic approach to injury, and riders often revel in physical risk rather than take steps to mitigate them, which may be similar to the attitude seen in horseracing staff here [75]. The drive to keep working through injury or pain could be seen as an embodied necessity, and stopping work undermines the social and cultural capital derived from the activity or engagement in that community [75,79,80]. The potential disengagement from injury discussion beyond the identification of injuries as a ‘list of accomplishments’ seen in this population warrants further consideration and is something researchers should factor into study design moving forwards when working with horseracing staff.

### 4.2. Injury Management

During the 12-month period, staff typically relied on pain management strategies to continue working whilst injured. Over half of staff reported to using over-the-counter medication at least once per week to manage daily tasks at work, with 18% taking painkillers daily. Only 10% of staff did not use any medication to manage physical pain at work. Use of prescription medication for pain relief has previously been reported in 4–8% of racing staff [10], although this was not categorized by drug type, or reason for use. The use of analgesics as a tool to comply with presenteeism is seen in several vocations, including sporting athletes [81]. There is a high reported use of non-steroidal anti-inflammatory drugs (NSAID’s) in sport [82,83] with Harle *et al.,* [84] suggesting over 50% of elite athletes use oral non-steroidal anti-inflammatory drugs (NSAIDs) at international events. Similarly, equestrian athletes report a high prevalence of taking medication, with 51% of dressage riders [58], 67% of show jumpers [57], and 96% of event riders [56] using pain relief medication during equestrian activities (training and competing). Overbye [81] suggests that the practice of using analgesics to maintain performance, improve recovery and reduce the impact of injury has become a socially accepted practice in sport, and is often encouraged by peers and coaches as “routine” despite potential negative side effects. When taken frequently, NSAID’s can cause side effects to the gastrointestinal system and kidneys [81,85], with some research suggesting long term analgesic use can result in negative effects on muscle recovery [86], which would further increase the risk of injury and may partially explain the higher rates of chronic pain observed in older racing staff [2]. The high reported use of painkillers within this study to maintain daily occupational demands highlights a potential problem with the overuse of pain medication in the racing industry and is something that requires further investigation into the long-term consequences on staff health.

Over half the racing staff surveyed here reported continuing to work without any adaptations or accommodations, whilst less than a quarter of staff took time off in relation to the injury sustained, similar to previous industry reports [4,8,10]. Of those who continued to work, adaptations to working environment mostly involved reduced duties or restrictions in ridden activity. Most workers with recurrent musculoskeletal pain or discomfort continue to work, reporting minimal time loss [87], however, this has previously been seen to decrease workplace productivity [88]. Due to concerns with staffing structure, and the decreased workforce retention seen in horseracing presently, staff who are ‘not pulling their weight’ may be seen to be an inconvenience or nuisance, which could result in loss of job security, which is already of concern to this population [10,17,34]. The inability to work at normal capacity, due to injury or fatigue, may also increase the risk of injury to horses under the care of stable staff or lead to poor management practices [18,19]. Lack of concentration, physical fatigue and burnout resulting from a stressful working environment or reduced mental resilience can negatively impact task efficiency by affecting visual acuity, accuracy, and individual reaction time [60]. Slower reactions, or loss of focus around horses could result in preventable injury to both parties or result in subpar management and care of the horse, thus highlighting the importance of a healthy workforce to maintain high equine welfare standards within the sector.

Despite concerns about presenteeism in horseracing staff, Crawford et al. [89] identify that staff are not required to be 100% fit to return to work, however, accommodations should be made, based on work ability, to facilitate a safe return, which was seen in a large group of participants in this study. Research suggests that workplace interventions can make return to work easier, enhance quality of life and reduce the cost of injuries to the healthcare industry [90]. Returning to work earlier can also result in improvements in physical recovery and has psychosocial benefits, such as enhanced social connection and heightened morale [91]. Return to work is often facilitated by temporary modifications to the job role [87], managed by the employer [22,23] and may be physical, social/organisational, or psychological [89]. This study saw a range of physical adaptations, such as changes in job requirements or restricted duties, as well as flexibility in changing work hours which are classified as organisational adaptations. Williams et al. [90] suggests that adaptations of both task requirements (physical) e.g., non-ridden duties or less boxes to muck out, and working hours (organisational) are effective at managing return to work from injury, whilst modification to the working environment overall has been shown to be effective at reducing the perception of pain. Modification of tasks is a common workplace adaptation following injury or illness [21,24] and has been linked to increased perception of job control, a factor that could mitigate the risk of re-injury and improve overall job satisfaction [23,24,36,61]. Whilst adaptations were reported in this study, the ability to temporarily modify job roles within horseracing would be dependent on managerial culture and staffing structure within the yard environment. Many training and stud yards are understaffed [17,67,68], increasing the difficulty to provide adequate workplace adaptations for injured staff. Trainers previously reported finding staff cover a substantial workplace stressor [9], whilst staff reported an increase in physical effort because of a diminished workforce [17,34]. Staff in this study felt guilty that colleagues were “carrying the weight” whilst they were injured, which may suggest this population would be unlikely to follow strict restrictions on workplace tasks if they were to be implemented. The staff shortage was further exacerbated by ongoing COVID-19 restrictions in place [23], whereby staff were under increased pressure to maintain high standards whilst adhering to social distancing requirements, hygiene protocols and covering staff who were isolating, shielding or unwell [23,34]. Where horseracing staff may continue to work whilst experiencing injury, or may return to work early, workplace interventions such as adaptations to tasks and hours, should be implemented as a standard protocol, with modifications implemented on an individual basis, considerate of physical limitations, injury type and pain levels. These adaptations should be monitored closely and return to work should be contingent on reduced workload subject to doctors’ approval.

Work friends, parents, and employers were viewed as both positive and negative support mechanisms whilst coping with injury, which reflects previous sport & occupational health literature [28,89,92]. Social support, such as from employers, friends, family, or colleagues, is particularly important in maintaining adherence to rehabilitation, and disengagement can lead to feelings of isolation, which decreases adherence to rehabilitation [93,94]. Udry et al. [95] suggested more athletes reported negative social support than positive, whilst 54% of unhelpful supporters are typically family members [96]. Comparatively, in military personnel, home-based social support (including family members) has been shown to be a protective factor for veterans at risk of suicide [97]. Interestingly, Tveito et al. [87] found that workers were concerned about being too vocal in complaints of pain at work due to fear of annoying colleagues, which has been seen here in this study, with staff reporting concern over “embarrassment”, “looking weak”, “being a nuisance” or “being seen as soft”. Research suggests that post-trauma, negative social support, such as criticism or indifference to the wellbeing of that person, has a greater impact on successful recovery outcomes than lack of support [28].

The quality of the relationship can influence perception of support more than the nature of the relationship between individuals [92], and open communication between employers and employees is recognized as critical to a successful return to work following injury by the World Health Organisation (WHO) [89]. Open communication is a key managerial skill, something reported as lacking within the horseracing sector, whereby staff are often promoted to management level due to horsemanship skills rather than people skills [17]. Management behaviour have also been found to be a key factor in influencing how employees handle pain at work [98,99]. Within the racing industry, 44% of employees previously stated that their employer was “not supportive at all” in response to their injury rehabilitation, which could affect rehabilitation success and recovery in stable staff, and mirrors the results shown in this study (41%) [10] (pp. 39). Trauma within the workplace can also create a distrust in senior staff, who are entrusted with care of employees and a sense of betrayal may be formulated here which can further affect communication between staff and employees and exaggerate the underreporting of work-based injuries previously seen in this population [7,9,10,28]. The disparity seen here in whether employers are supportive during the injury process may stem from ineffective people management practices in horseracing yards, and highlights a need for specialized, targeted managerial training to support staff who are promoted to senior management [17,24].

Under the U.K. national legislative framework, the employer has a responsibility to manage employee risk at work, including taking measures to control risk, therefore managerial or senior staff are critical in successful injury management within racing yards [23]. Whilst there has been an increasing amount of guidance available for employers on mental health in the workplace in recent years, there is still a lack of guidance on musculoskeletal pain and injury available [89]. Palsson et al. [100] identified that educational resources for organisations could reduce pain-related loss of workability and fostering cultures of open communication with staff could decrease absenteeism in the workforce, whilst both training and targeted organizational campaigns were found to have a positive effect on injury reduction globally [24]. The Racing Occupational Health Service offers workplace talks as part of their suite of services [26], however, these resources are relatively new within the industry, and according to this survey are underutilized. Furthermore, as OHS provision is voluntary within the U.K., the responsibility for promotion of available services lies with the employer, suggesting that racing staff may not be accessing Racing Welfare’s suit of services as they are not being directed to them by their employer when required. Further development of Racing Welfare’s educational resources, particularly targeted to employers to highlight the economic, performance and productivity benefits for OHS provision in the workplace [21,22,23,24] would be greatly beneficial to maintaining the health and wellbeing of the workforce in horseracing.

### 4.3. Attitudes to Injury and Injury Reporting Behaviours

The results of this study indicate presenteeism is seen in horseracing, and there was a tendency for staff to underreport occupational injuries to their employer. Attitudes towards injury reporting behaviours in this study were influenced by a range of sociocultural and occupational factors, including perceived industry expectations, financial circumstances, previous experiences of injury, staffing structure and perceived employer expectations.

Injury reporting has previously been considered a concern in the racing industry, with anecdotal reports of staff unwilling to take sick leave or continuing to work despite chronic pain or injury [2,7,9,10]. Staff often cite a love of the job, moral or ethical obligations (for example to animal welfare), or concerns for job security as reasons for not taking adequate time off [10,101]. Underreporting of injuries, or not seeking subsequent medical intervention, has also been seen in wider equestrian sports [102], whereby injury is seen as something that cannot be avoided but should not delay or prevent engagement with equestrian activities [75], suggesting there may be a cultural connotation with injury attitudes in horse-related industries, rather than solely within horseracing. In other animal care industries, presenteeism is often associated with guilt, as well as concern that animal welfare is being impacted by their absence [18], resulting in continuing to work despite injury or illness. Within horseracing, the requirements to maintain high standards of care of the horses is vital for the success of training yards, and if injured staff prioritize their own needs and career ahead of daily management and care of the horses in their care, they may experience guilt, linked to both equine welfare and perhaps their colleagues who are taking on additional work.

Employees may also reduce reporting behaviour to avoid guilt for letting the team down, which has been seen in injured athletes [103,104,105]. Within the racing industry, there is currently a staff shortage, which can lead to issues with being covered if off sick or injured [9,67,68]. Different to the psychological belief that an employee is irreplaceable [18], the current working conditioning within racing highlights a physical lack of staff who can cover shifts. This was highlighted as a concern for trainers in Sear’s [9] study, whereby finding staff cover was reported as a main source of stress for those working in industry. Injury has been previously highlighted as a significant source of stress for managerial or coaching staff, who are in positions of responsibility to ‘fill the gaps left by injury’ within a team, much the same as a trainer [9,106]. If this stress is made known, directly, or indirectly, to a team of subordinates, that team may alter their behaviours, and subsequently hide injuries or pain, to reduce stress on their manager, particularly where good relationships have been developed. Many staff in this study highlighted a pressure to continue working, either related to their employer (don’t want to hassle them, not necessary), to other staff (burden on other staff) or to the horses themselves (horses need me).

However, in this study, some staff did identify a clear injury reporting protocol, and stressed the importance of following this in their own practice (and influencing others to do the same) to maintain staff health and wellbeing. In both horseracing and equestrian sport, horsemanship skills are typically learnt in apprenticeship positions [20] and in deference towards those with greater equine experience [17,75], thus attitudes to injury are often ‘taught’ through peer-to-peer interaction [107]. This could suggest that whilst injury minimalization culture is a concern in horseracing [16], its prevalence and impact on injury reporting may be subject to individual yard microcultures, rather than a comprehensive industry-wide problem. Further research should consider the role of individual yard culture on injury reporting practices, and design educational intervention packages to reduce the stigma associated with injury and increase awareness of the implications of injury denial on employee health and wellbeing.

Whilst there are many factors that can affect reporting behaviour in occupational settings, an increase in injury severity typically results in improved reporting behaviours [107], however, that relationship is not seen here with respect to concussion. Staff were more likely to seek medical attention and report injuries to their employer for visible injuries, such as fractures, compared to suspected concussion or other musculoskeletal injuries. Underreporting of concussion has been identified as a common issue in athletic populations, and it is believed that many sport concussions go unreported and undiagnosed due to limited disclosure of symptoms from athletes [108,109]. Common reasons for hiding symptoms includes downplaying severity, loss of athletic standing amongst coaches or peers [108] and prior experiences of concussion resulting in belief of ‘knowing oneself’ and limits of capability linked to the current concussion [109]. Downplaying the severity of injuries could be attributed to injury denial [110,111], which is one of the five stages of grief commonly attributed to athlete injury during the emotional response phase [37]. Denial often results in emotional instability following injury [93], and difficulty coping with stress [112], which could result in risk of reinjury, as well as impact workplace retention and career longevity.

Horseracing has previously been at the forefront of concussion protocols for jockeys since 2003 with the introduction of the British Horseracing Authority’s (BHA) standardized concussion protocol [20], however, this study would suggest that the self-management of concussion for staff on training and stud yards is suboptimal. Whilst most research suggests the need to enhance concussion education for athletes and coaches, or in this instance, staff and trainers [10,109], Conway et al. [108] suggested that there was no relationship between lack of concussion knowledge in athletes and underreporting behaviours, in fact higher knowledge often related to enhanced ability to hide symptoms. National targeted media strategies highlighting the reasons why athletes should disclose concussion, as well as the implications for non-disclosure on health, finances, and support networks (family, friends, spouse, children) is equally important to increasing reporting behaviours in sport [108]. Horseracing has previously used several major national campaigns to promote healthy behaviour in professional jockeys, such as the 2016 Jockey Matters campaign run by the Jockey Education and Training Scheme (JETS) [113], which provided educational resources and helplines offering support on nutrition, physical fitness, injury and concussion and mental health. Stable staff recently highlighted the need for such resources [10] despite some already being provided through Racing Welfare. A national campaign to promote concussion awareness, alongside standard protocols for stud and stable staff are the next steps for the industry to tackle workplace concussion.

Within this study, women were less likely to report musculoskeletal injuries to their employer, as well as less likely to seek medical attention and take time off compared to male counterparts. Wider research in Sweden has identified that the highest rates of underreporting occupational injuries were seen in female dominated organisations [107], whilst culturally, men are more likely to engage in risk taking behaviour which is causally linked to injury incidence [78]. Male athletes have also been found to affiliate with hypermasculine ideologies, such as indifference to physical pain, which has been found to be a predictor in negative attitudes to seeking help [114]. Within the racing industry, significant strides have been made regarding gender equality, with current estimations of a 70:30 split (female to male) on racing yards [115], however, there are still some residual perceived sex imbalances which can act as key barriers for women in racing to advance in the industry [115,116,117]. As a result of the potential gender biases within the workforce culture in racing, female staff may be less likely to report injuries compared to their male counterparts for fear of being viewed as weaker [117]. Employers within the racing industry should be conscious that female staff may be more likely to report injury behaviours differently to their male counterparts [107].

### 4.4. Limitations

There are limitations to consider within the study. The online sample, although a quick way to obtain access to a wider population of staff, may have been subject to self-selection bias [118]. As a result, responses may be skewed to include only those staff who were significantly impacted by injury, thus wider generalization should be considered with caution. In addition, despite significant industry promotion and support to publicize the survey, including strong engagement with social media posts, a smaller percentage of the eligible population of racing staff engaged with the survey than expected. Injury reporting is considered a concern in the racing industry, and these attitudes have been discussed earlier in this paper [2,7,9,10]. Under-reporting of injury is a common occurrence in other vocations as well, for example, 49–58% of musculoskeletal injuries go unreported in the military, and often attributed to an injury minimalization culture [12]. Racing research has previously reported that staff conform to an organisation’s expectations of a job role, through the suppression and regulation of emotional displays that do not meet typically “tough” characteristics racing staff are expected to adhere to, such as during periods of injury or pain [2,9]. Cassidy [3] suggested that horseracing may be an example of an organisational culture where the employees act, think and feel in accordance with cultural expectations, and new staff entering are taught to adhere to those cultural norms. In horseracing, as seen in the results of this study, such cultural norms may include acting tough, working in pain, hiding injuries, or hiding emotions for fear of losing respect or their job. This perceived culture could influence the likelihood of honest reporting of injury experiences, seen in the drop-out rate early in the questionnaire, which could hinder further research in this field.

### 4.5. Recommendations and Future Research Directions

Given the findings of this study, several recommendations and future directions are proposed. Stud and stable staff reported the highest rates of injury within this study, and were a key risk group for underreporting injury, and presenteeism. Further research should explore the personal injury experiences of racing grooms, considering the four themes identified in this study (physical, mental, occupational and lifestyle implications). In addition, this study has highlighted wider sociocultural viewpoints on injury normalization which may be influencing individual management practices, injury reporting behaviour, and overall staff wellbeing. Further research should look to examine whether there is an injury minimalization culture within horseracing staff and the effects this may have on injury management within stud and training yards. Recommendations from this study include:

1. An online Horseracing Occupational Health & Injury Training Package for stud and stable staff, highlighting risk factors for injury, management of equipment use during repetitive tasks (such as tools for mucking out and sweeping), and early predictors of musculoskeletal injury to raise awareness and promote positive self-management of physical health and wellbeing. Given racing staff’s workload and time constraints, the training package is recommended to be online and shorter in length (i.e., 1 h);
2. The development and subsequent implementation of a national return-to-work procedure for injured stud and stable staff. Employment modifications and workplace adaptations implemented on an individual basis following discussions with line manager, considerate of physical limitations, injury type and pain levels;
3. Targeted senior staff and managerial training on occupational injury and injury management, embedded into pre-existing courses run through the British Racing School, such as the Yard Managers Course, or the Trainers Modules (1–3) which are a requirement to hold a Trainers License in Great Britain [20];
4. The expansion of the Racing Occupational Health Service and its resources, including targeted educational resources on musculoskeletal injury and pain available for horseracing organisations and employers;
5. A national campaign to promote concussion awareness in stud and stable staff within the horseracing industry, which would aim to increase concussion knowledge, reduce stigma of reporting and direct staff to appropriate support services. To follow, the development of standardized protocols for concussion management in yard settings to replicate industry excellence seen in jockey concussion protocols.

## 5. Conclusions

Occupational health of stable staff is a key priority for the racing industry, and it is becoming increasingly more important to consider the nature of injury for stable staff, in line with the British Horseracing Authority (BHA) Recruitment, Training and Retention strategy. The present study is one of the first to identify occupational risk factors for horseracing staff, beyond the horse itself, as well as to identify psychological and sociocultural effects of occupational injury in horseracing staff. Staff reported a high rate of injuries, with working hours, perceived job security and perceived job control factors in resultant injury type. Physical limitations, loss of confidence, workplace changes, and lifestyle implications were reported as the key areas affected by personal injury and further research is warranted to explore the personal injury experiences of racing staff. Staff were less likely to report or take time-off for invisible injuries, such as concussion or musculoskeletal pain, compared to fractures, and attitudes towards injury reporting and management were influenced by financial circumstances, perceived staff shortages, previous injury experiences, and perceived employer expectations. The industry attitudes towards injury reporting and management seen in this study may provide opportunities to influence workplace safety within horseracing, through the development of education and training packages for staff and employers, aimed at reducing stigma, and improving knowledge of injury implications, particularly linked to the long-term implications of concussion in the workforce.

## Figures and Tables

**Table 1:** Questionnaire topic areas [7,9,16,34].

Topic Area	Key Focus	Justification
Demographics	Age, biological sex, years in industry, geographical location (region)	Discrepancies in injury prevalence between age groups and gender seen in previous research [4,7,8,35].
Employment characteristics	Job type, full or part time contract, hours, pay, job control	Job characteristics and limited job control is a key factor in work-based injury and stress [36]. Part time or casual staff more likely to suffer increased injuries [13,14].
Injury Characteristics	Injury type, incidence, experience of injury	Injury causation and situational context are factors that may affect cognitive appraisal of the injury [37], changing emotional responses and rehabilitation/coping behaviours [38,39].
Injury management and attitudes to injury (including coping)	Approaches to injury management (personal and professional), pain management practices, support networks	Under-reporting is an anecdotal concern for the racing industry. Institutional habitus and expectations of ‘toughness’ seen in racing staff [2,3,40].

**Table 2:** Categorization of staff by racing code.

Code	N	Percentage Total (%)
Dual (Flat and Jump)	63	32.1
Flat Racing only	52	26.5
Jump Racing only	52	26.5
Point-to-point racing	8	4.0
Arabian racing	1	0.5
Other	20	10.2

**Table 3:** Number of years working in horseracing.

Number of Years	N	Percentage Total (%)
1–5 years	63	32.1
6–10 years	45	23
11–15 years	26	13.3
16–20 years	20	10.2
21–25 years	14	7.1
26+ years	28	14.3

**Table 4:** Average working hours.

Hours (per day)	N	Percentage Total (%)
1–3 h	11	5.6
4–5 h	32	16.3
6–7 h	50	25.5
8–9 h	66	33.67
10–11 h	25	12.76
12+ h	12	6.1

**Table 5:** Workplace injuries reported by horseracing staff in the last 12 months.

Injuries	N	Percentage Total (%)
Bruises	283	23.5%
Lower back pain (lumbar)	175	14.5%
Muscle strain	163	13.5%
Upper back or neck pain (cervical/thoracic)	105	8.7%
Lacerations	72	6%
Tendon/Ligament damage	63	5.2%
Concussion (suspected)	62	5.1%
No Injuries in the last 12 months	42	3.5%
Sprained ankle	39	3.2%
Rib bruising or rib fractures	36	3%
Fractures—arms or hand	31	2.6%
Concussion (diagnosed by clinician)	23	1.9%
Sprained wrist	22	1.8%
Fractures—leg or foot	20	1.7%
Nerve damage	19	1.6%
Other	16	1.3%
Dislocation e.g., shoulder or knee	15	1.2%
Other head injuries	11	0.9%
Fractures—spine	6	0.5%
Fractures—skull	3	0.3%

**Table 7:** Perceptions of how helpful key support people are during periods of injury.

Person	Unhelpful (%)	Helpful (%)	Not Applicable (%)
Work friends	33.34	52.22	14.44
Parents	22.22	47.78	30.00
Employer	41.11	45	13.89
Spouse	15.88	42.78	41.67
Family	19.45	19.44	61.11
Children	10	9.44	80.56
Other	7.22	7.22	85.56

## Data Availability

Data are confidential thus not publicly available for access.

## Supplementary Materials

The following supporting information can be downloaded at: https://www.mdpi.com/article/10.3390/ijerph19042054/s1, File S1: Questionnaire.
