# Supplementary material for: Is Injury an Occupational Hazard for Horseracing Staff?
Source: Int J Environ Res Public Health. 2022 Feb 12;19(4):2054. doi: 10.3390/ijerph19042054 (PMC8871636; doi:10.3390/ijerph19042054)
Supplement: Supplementary file 1 [file ijerph-19-02054-s001.zip › ijerph-1554645-supplementary.pdf]

Supplementary File S1 – Questionnaire

Q1 Have you experienced any of the following injuries in the last 12 months, that were caused by working directly with horses in the breeding and racing industry? (Tick all that apply)

- ▢ Bruises (1)
- ▢ Lacerations (2)
- ▢ Fractures - arm or hand (3)
- ▢ Fractures - leg or foot (4)
- ▢ Dislocation e.g. shoulder, knee etc. (5)
- ▢ Sprained wrist (6)
- ▢ Sprained ankle (7)
- ▢ Muscle strain (8)
- ▢ Tendon/ligament damage (9)
- ▢ Rib bruising or rib fractures (10)
- ▢ Upper back or neck pain (cervical/thoracic) (11)
- ▢ Lower back pain (lumbar) (20)
- ▢ Fractures - spine (12)
- ▢ Concussion (suspected) (19)
- ▢ Concussion (diagnosed by clinician) (13)
- ▢ Fractures - skull (14)
- ▢ Other head injuries (15)
- ▢ Nerve damage (16)
- ▢ Other, please state (17) ____
- ▢ No Injuries in the last 12 months (18)

Skip To: Q7 If Have you experienced any of the following injuries in the last 12 months, that were caused by wor... = No Injuries in the last 12 months

Q2 In the last 12 months, were you required to make any of the following adaptations to your working life because of injury? Please select all that apply.

- ▢ Decreased number of working hours per day (4)
- ▢ Reduced days per week (13)
- ▢ Required to stop work (please state how long for in weeks) (16)

____

- ▢ More rest breaks during the day (5)
- ▢ Not allowed to ride (6)
- ▢ Permitted to ride, but restrictions on number of lots or type of horse (12)
- ▢ Reduced duties (non-riding) (7)
- ▢ I made no adaptations to my working life based on injury (14)
- ▢ Other (please state) (8) ____

Q3 If you have any comments about how injury has affected you in the last 12 months, please outline them here.

____

Q4 Please highlight how helpful the following people were during the times you were injured in the last 12 months:

	Extremely unhelpful (1)	Mostly unhelpful (2)	Neither helpful nor unhelpful (3)	Mostly helpful (4)	Extremely helpful (6)	Not applicable (7)
Spouse/Partner (1)	○	○	○	○	○	○
Parents (2)	○	○	○	○	○	○
Children (3)	○	○	○	○	○	○
Other Family Members (4)	○	○	○	○	○	○
Work friends (5)	○	○	○	○	○	○
Employer (6)	○	○	○	○	○	○
Other, please state (7)	○	○	○	○	○	○

Q5 Have you accessed any of the following services for support or advice (online, in person or over the phone) due to injury in the last 12 months? Please select all that apply.

- ▢ Arabian Racing Organisation (13)
- ▢ British Horseracing Authority (BHA) (6)
- ▢ Injured Jockeys Fund (IJF) (3)
- ▢ National Association of Racing Staff (NARS) (2)
- ▢ National Trainers Federation (NTF) (5)
- ▢ NHS (12)
- ▢ Racing's Occupational Health Service (9)
- ▢ Racing Welfare (1)
- ▢ Thoroughbred Breeders Association (TBA) (11)
- ▢ Other, please state (7) ____
- ▢ I have not used any support services for my injuries (8)

Q6 How frequently have you seen staff (yourself or those working around you) experience the following injuries working in the racing industry?

	Daily (1)	Weekly (2)	Monthly (3)	Every 3 months (4)	Every 6 months (5)	Once per year (6)	Not in the last 12 months (7)
Any Fracture (1)	○	○	○	○	○	○	○
All other musculoskeletal injuries e.g. Anterior Cruciate Ligament (ACL) injury, ankle sprain etc. (2)	○	○	○	○	○	○	○
Concussion (diagnosed or suspected) (4)	○	○	○	○	○	○	○
Other Head injuries e.g. brain bleed etc. (3)	○	○	○	○	○	○	○

Carry Forward All Choices - Displayed & Hidden from "How frequently have you seen staff (yourself or those working around you) experience the following injuries working in the racing industry? "

Q7 For each of the injuries listed, identify the likelihood that you would take any of the following actions:

	Seeking Medical Attention beyond immediate First Aid (i.e. visit doctor, A&E, minor injuries unit etc.)					Reporting the Injury to your employer					Taking time off work as a result of the injury				
	Extremely Unlikely (1)	Somewhat Unlikely (2)	Unsure (3)	Somewhat Likely (4)	Extremely Likely (5)	Extremely Unlikely (1)	Somewhat Unlikely (2)	Unsure (3)	Somewhat Likely (4)	Extremely Likely (5)	Extremely Unlikely (1)	Somewhat Unlikely (2)	Unsure (3)	Somewhat Likely (4)	Extremely Likely (5)

Arm Fractures (x1)	○	○	○	○	○	○	○	○	○	○	○	○
Dislocations (x2)	○	○	○	○	○	○	○	○	○	○	○	○
Concussion (x4)	○	○	○	○	○	○	○	○	○	○	○	○
Sprained ankle (x3)	○	○	○	○	○	○	○	○	○	○	○	○

Q8 Briefly explain your answers to the previous question.

____

Q9 On average, how often do you need to take over the counter pain medication (i.e. ibruprofen or paracetamol) to complete your daily tasks at work?

- ○ More than once a day (6)
- ○ Daily (11)
- ○ 2-3 times a week (8)
- ○ Once a week (9)
- ○ Once a month (10)
- ○ Once every 3 months (12)
- ○ Once every 6 months (13)
- ○ Once a year (14)
- ○ Less than once a year (15)

Q10 In the last 12 months, have you ever used any of the following substances to manage physical pain related to a work-based injury? (Please select all that apply)

- ▢ Over-the-counter pain medication (1)
- ▢ Alcohol (2)
- ▢ Nicotine (smoking, vaping etc.) (3)
- ▢ Prescription pain killers (with a specific prescription) (4)
- ▢ Prescription pain killers (without a specific prescription) (5)
- ▢ Illicit substances i.e. cocaine, cannabis etc. (6)
- ▢ Legal highs i.e. spice, legal E etc. (7)
- ▢ I have used none of these substances for pain management in the last 12 months (8)
- ▢ Prefer not to say (9)
- ▢ Other (please state) (10) ____

Q11 What is your current employment status?

- ○ Full time (1)
- ○ Part time (2)
- ○ Self-employed (3)
- ○ Director Ltd. Company (8)
- ○ Incapacitated/long term sick leave (9)
- ○ Other, please state (4) ____

Q12 Please select the option which best represents the number of years you have been employed in racing (either full or part time):

- ○ 1 - 5 years (1)
- ○ 6 - 10 years (2)
- ○ 11 - 15 years (3)
- ○ 16 - 20 years (4)
- ○ 21 - 25 years (5)
- ○ 26 years + (6)

Q13 Please indicate which code you primarily work within:

- ○ Flat racing (1)
- ○ National Hunt racing (2)
- ○ Both (3)
- ○ Point-to-Point (5)
- ○ Arabian Racing (6)
- ○ Other, please state (4) ____

Q14 What best represents your current industry role?
General Area (1)
Specific Role (2)

- ○ Racecourse (1)
- ○ Racecourse ~ Stalls Handler (2)
- ○ Racecourse ~ Admin (3)
- ○ Racecourse ~ Catering (4)
- ○ Racecourse ~ Grounds Staff (5)
- ○ Racecourse ~ Operations (6)
- ○ Racecourse ~ Security & Integrity (7)
- ○ Auxillary (8)
- ○ Auxillary ~ Box Driver (9)
- ○ Auxillary ~ Farrier (10)
- ○ Auxillary ~ Gallops Man (11)
- ○ Auxillary ~ Valet (12)
- ○ Breeding (13)
- ○ Breeding ~ Admin/Secretary (14)
- ○ Breeding ~ Bloodstock Agent (15)
- ○ Breeding ~ Grounds Staff (16)
- ○ Breeding ~ Maintenance (17)
- ○ Breeding ~ Sales Staff (18)
- ○ Breeding ~ Stallion Man (19)
- ○ Breeding ~ Stud Hand (20)
- ○ Breeding ~ Supervisory (21)
- ○ Breeding ~ Young Learner (22)
- ○ Training Yards (23)

- Training Yards ~ Admin/Secretary (24)
- Training Yards ~ Arab Racing (25)
- Training Yards ~ Jockey (26)
- Training Yards ~ Maintenance (27)
- Training Yards ~ Point to Point (28)
- Training Yards ~ Pre-Training/Rehab (29)
- Training Yards ~ Rider/Groom (30)
- Training Yards ~ Supervisory (31)
- Training Yards ~ Trainer (32)
- Training Yards ~ Travelling Groom (33)
- Training Yards ~ Yard Person (34)
- Training Yards ~ Young Learner (35)

Q15 Please indicate how long your average working day is:

- 1 - 3 hours (1)
- 4 - 5 hours (2)
- 6 - 7 hours (3)
- 8 - 9 hours (4)
- 10 - 11 hours (5)
- 12 hours + (6)

Q16 Which of the following statements best describes your feelings towards your average working hours?

- I am satisfied with the hours I work (1)
- I work too few hours and would like to work more (2)
- I work too many hours and would like to work less (3)

Q17 On a scale of 1 to 5, how much control do you have over managing how your daily tasks are organised? 0 = no control, 5 = complete control

- No Control (1)
- A little Control (2)
- A moderate amount (3)
- A lot of control (4)
- All tasks are under my control (5)

Q18 How secure is your current role in the industry? (i.e. would you still be employed in 12 months' time if you so wished to remain)

- ○ Definitely secure (1)
- ○ Probably secure (2)
- ○ Unsure (3)
- ○ Probably not secure (4)
- ○ Definitely not secure (5)

Q19 In the last 12 months, have you ever used any of the following substances <u>socially</u>? Please select all that apply.

- ☐ Over-the-counter pain medication (1)
- ☐ Alcohol (2)
- ☐ Nicotine (smoking, vaping etc.) (3)
- ☐ Prescription pain killers (with a specific prescription) (4)
- ☐ Prescription pain killers (without a specific prescription) (5)
- ☐ Illicit substances i.e. cocaine, cannabis etc. (6)
- ☐ Legal highs i.e. spice, legal E etc. (7)
- ☐ I have used none of these substances socially in the last 12 months (8)
- ☐ Prefer not to say (9)
- ☐ Other (please state) (10) ____

Q20 On average, how many units of alcohol do you drink per week? Please refer to the image for guidance.

- ○ I do not drink alcohol (1)
- ○ 1 - 5 units (2)
- ○ 6 - 10 units (3)
- ○ 11 - 15 units (4)
- ○ Over 15 units (5)

Q21 Tick the box that best represents how you have been feeling (in the last week) in response to each statement given below:

	Not at all (1)	Sometimes (2)	Often (3)	Very Often (4)
I feel tense or 'wound up' (1)	○	○	○	○
I get a sort of frightened feeling as if something awful is about to happen (2)	○	○	○	○
Worrying thoughts go through my head (3)	○	○	○	○
I can sit at ease and feel relaxed (4)	○	○	○	○
I get a sort of frightened feeling like 'butterflies' in the stomach (5)	○	○	○	○
I feel restless like I have to be on the move (6)	○	○	○	○
I get sudden feelings of panic (7)	○	○	○	○
I still enjoy the things I used to enjoy (8)	○	○	○	○
I can laugh and see the funny side of things (9)	○	○	○	○
I feel cheerful (10)	○	○	○	○
I feel as if I am slowed down (11)	○	○	○	○
I have lost interest in my appearance (12)	○	○	○	○
I look forward with enjoyment to doing things (13)	○	○	○	○
I can enjoy a book, TV show or the radio (14)	○	○	○	○

Q22 On average, how often do you place bets on sporting events? N.B. This can be on any sport, using phone, mobile or web-based betting sites, or live at an event.

- Daily (1)
- 2-6 times a week (2)
- Once a week (3)
- 2-3 times per month (4)
- Once a month or less (5)
- I do not bet (6)

Q23 In the last 12 months, how often have you...

	Never (1)	once a month or less (2)	2 - 3 times per month (3)	once per week (4)	2 - 6 times per week (5)	Once a day or more (6)
Gone on eating binges where you feel that you may not be able to stop? (1)	○	○	○	○	○	○
Made yourself sick (vomited) to control your weight or shape? (2)	○	○	○	○	○	○
Used laxatives, diet pills or diuretics (water pills) to control your weight or shape? (3)	○	○	○	○	○	○
Exercised more than 60 minutes a day (outside of racing activities/riding) to lose or to control your weight? (4)	○	○	○	○	○	○

Q24 Please select your gender

- ○ Male (1)
- ○ Female (2)
- ○ Transgender (3)
- ○ Other, please state (4) ____________________

Q25 What is your current age?

____

Q26 Please tick the geographical location that best represents where you work:
Country (1)
Region (2)

- ○ England (1)
- ○ England ~ North West (2)
- ○ England ~ North East (3)
- ○ England ~ Yorkshire & the Humber (4)
- ○ England ~ East Midlands (5)
- ○ England ~ West Midlands (6)
- ○ England ~ East (7)
- ○ England ~ London (8)
- ○ England ~ South East (9)
- ○ England ~ South West (10)
- ○ Scotland (11)
- ○ Scotland ~ Borders (12)
- ○ Scotland ~ Central (13)
- ○ Scotland ~ Highlands & Islands (14)
- ○ Wales (15)
- ○ Wales ~ North (16)
- ○ Wales ~ Mid (17)
- ○ Wales ~ South (18)

Thank you for completing our survey.

If you feel you have been impacted by an injury, or any of the questions asked within this survey, please seek professional support (physical or psychological) using some of the resources provided below.

Local GP: https://www.nhs.uk/service-search/find-a-gp
Racing Welfare: https://racingwelfare.co.uk/services/info-advice-guidance/accidents/
Racing Welfare & Occupational Health: https://racingwelfare.co.uk/racings-occupational-health-service/
